# Weighted Change-Point Method for Detecting Differential Gene Expression in Breast Cancer Microarray Data

**DOI:** 10.1371/journal.pone.0029860

**Published:** 2012-01-20

**Authors:** Yao Wang, Guang Sun, Zhaohua Ji, Chong Xing, Yanchun Liang

**Affiliations:** 1 Key Laboratory for Symbol Computation and Knowledge Engineering of National Education Ministry, College of Computer Science and Technology, Jilin University, Changchun, China; 2 Department of Breast and Thyroid Surgery, China-Japan Union Hospital, Changchun, China; 3 Department of Communication Engineering, Jilin University, Changchun, China; 4 College of Computer Science and Technology, Inner Mongolia Normal University, Huhhot, China; 5 Guanghua College of Changchun University, Changchun, China; Memorial Sloan Kettering Cancer Center, United States of America

## Abstract

In previous work, we proposed a method for detecting differential gene expression based on change-point of expression profile. This non-parametric change-point method gave promising result in both simulation study and public dataset experiment. However, the performance is still limited by the less sensitiveness to the right bound and the statistical significance of the statistics has not been fully explored. To overcome the insensitiveness to the right bound we modified the original method by adding a weight function to the *D_n_* statistic. Simulation study showed that the weighted change-point statistics method is significantly better than the original NPCPS in terms of ROC, false positive rate, as well as change-point estimate. The mean absolute error of the estimated change-point by weighted change-point method was 0.03, reduced by more than 50% comparing with the original 0.06, and the mean FPR was reduced by more than 55%. Experiment on microarray Dataset I resulted in 3974 differentially expressed genes out of total 5293 genes; experiment on microarray Dataset II resulted in 9983 differentially expressed genes among total 12576 genes. In summary, the method proposed here is an effective modification to the previous method especially when only a small subset of cancer samples has DGE.

## Introduction

Selecting differentially expressed genes [1], [2] is one of the most important tasks in microarray applications. Many methods were proposed to compare patterns of gene expression between cells or tissues of different kinds and under different conditions, for example, between normal and cancer cells. The goal of these methods has been to enable faster, simpler, more sensitive and systematic analyses [3]. Among these methods, t-statistics is a classical and widely-used DGE detecting methods, which works on the hypothesis that all the cancer samples are over-expressed compared with the normal samples [4]. Several other methods are also based on this hypothesis, such as empirical Bayes approach [5], mixture model approach [6], and SAM [7]. However, considering the heterogeneity of gene activation, many genes show increased expressions in disease samples, but only for a small number of those samples [8]. The study of Tomlins et al. [9], [10] shows that t-statistics has low power in this case, and they introduced cancer outlier profile analysis (COPA) method which performs better than the traditional t-statistics for cancer microarray data sets. More recently, several progresses have been made in this direction with the aim to design better statistics to account for the heterogeneous activation pattern of the cancer genes, such as non-parametric method PPST (permutation percentile separability test) [11] (Lyons-Weiler, 2004) and LRS (likelihood ratio test) [12] (Hu, 2008); percentile based methods OS (outlier sum) [13] (Tibshirani, 2007), ORT (outlier robust t-statistics) [14] (Wu, 2007) and TriORT [15] as an improvement to ORT; MOST (maximum ordered subset t-statistics) [16] (Lian, 2008) and TriMOST [17], which is an improvement to MOST.

Previously, we proposed a non-parametric change point statistics (NPCPS) method [18] based on modified Kolmogorov statistic to detect the single change-point (CP) in a data sequence [19]. This method compares directly the data distribution of normal and cancer group to detect conveniently the existence of possible change-point in the cancer group, giving an estimate of the change-point as well. Besides, as a non-parametric inferential method, NPCPS does not make assumptions about the probability distributions of the variables being assessed, and accordingly, it is not necessary to normalize the microarray data before calculating the test statistic like other parametric methods usually do. By simulation and experiment, NPCPS is effective for DGE detection and outperforms the compared methods with better ROC results in many circumstances [18]. However, the performance of this change-point based method is still limited by the less sensitiveness to the right bound and the statistical significance of the static has not been fully explored. Therefore, here we present an improved method, Weighted Change-Point Statistics (WCPS) aiming to break the limitations.

## Results and Discussion

### Monte Carlo simulation and ROC analysis

Monte Carlo simulation was applied to evaluate the hypothesis test used in the proposed method. For each Monte Carlo simulation, the proposed method was applied to an artificial 7000-gene dataset in normal distribution (mean = 0, standard deviation = 1) and multiple simulations were carried out with positive μ = 2, and different sample size *n* (normal group size *n*_1_ and cancer group size *n*_2_ equal to *n*/2) and DGE sample size *k* (0<*k*<*n*_2_). The false positive rate (FPR, i.e. genes with DGE were recognized as no DGE existence) and average estimate of change point (Table 1 and Table 2) were computed. Generally, for both methods, the estimate of change point and FPR enhanced together when *k* increased; after FPR dropped below the significance level (0.01 in this case), the estimated position converges to the actual position. However, for a given *k*, the proposed method outperforms the original NPCPS with closer CP estimate and lower FPR; with *k* increasing, the proposed method converged faster to the true change point and reached zero FPR before the original NPCPS method. For normally distributed data, between WCPS and NPCPS, the FPR is 0.09 versus 0.17; for skew-normally distributed data, the FPR was 0.08 versus 0.12. Besides, the mean absolute error (MAE) of estimated CP by WCPS was 0.03, while MAE by NPCPS was more than 0.06.

**Table 1:** CP estimate and FPR on data in normal distribution of size n1 = n2 = 25 with positive μ = 2.

k	Actual CP	Average estimate of CP		FPR with C(0.01) = 1.628	
		WCPS	NPCPS	WCPS	NPCPS
1	0.98	0.84	0.60	0.65	0.84
3	0.94	0.92	0.80	0.14	0.48
5	0.90	0.89	0.84	0.04	0.16
7	0.86	0.84	0.82	0.02	0.04
9	0.82	0.81	0.81	0.002	0.01
12	0.76	0.75	0.74	0.0	0.002
15	0.70	0.70	0.69	0.0	0.0
20	0.60	0.60	0.60	0.0	0.0
25	0.50	0.51	0.51	0.0	0.0
----	----	**MAE = 0.03**	**MAE = 0.07**	**Mean FPR = 0.09**	**Mean FPR = 0.17**

**Table 2:** CP estimate and FPR on data in normal distribution of size n_1_ = n_2_ = 50 with positive μ = 2.

k	Actual CP	Average estimate of CP		FPR with C(0.01) = 1.628	
		WCPS	NPCPS	WCPS	NPCPS
1	0.99	0.84	0.62	0.62	0.80
4	0.96	0.92	0.88	0.07	0.27
7	0.93	0.91	0.90	0.01	0.04
9	0.91	0.90	0.89	0.003	0.01
12	0.88	0.87	0.87	0.0	0.0
17	0.83	0.83	0.82	0.0	0.0
22	0.78	0.78	0.78	0.0	0.0
30	0.65	0.70	0.65	0.0	0.0
50	0.50	0.50	0.51	0.0	0.0
----	----	**MAE = 0.03**	**MAE = 0.06**	**Mean FPR = 0.08**	**Mean FPR = 0.12**

Results of more simulations with different μ and *k* are in Table 3.

**Table 3:** CP estimate and FPR on data in normal distribution of size n_1_ = n_2_ = 25 with different μ and *k*.

μ	k	Actual CP	Average estimate of CP	FPRC(0.01) = 1.628
−4	2	0.96	0.95	0.01
−3	4	0.92	0.93	0.01
−2	4	0.92	0.91	0.11
−2	3	0.94	0.92	0.18
−1	9	0.82	0.82	0.32
−1	5	0.90	0.85	0.44
3	4	0.92	0.93	0.01
3	5	0.90	0.91	0
4	3	0.94	0.95	0.01
4	5	0.90	0.91	0

**Figure 1:**
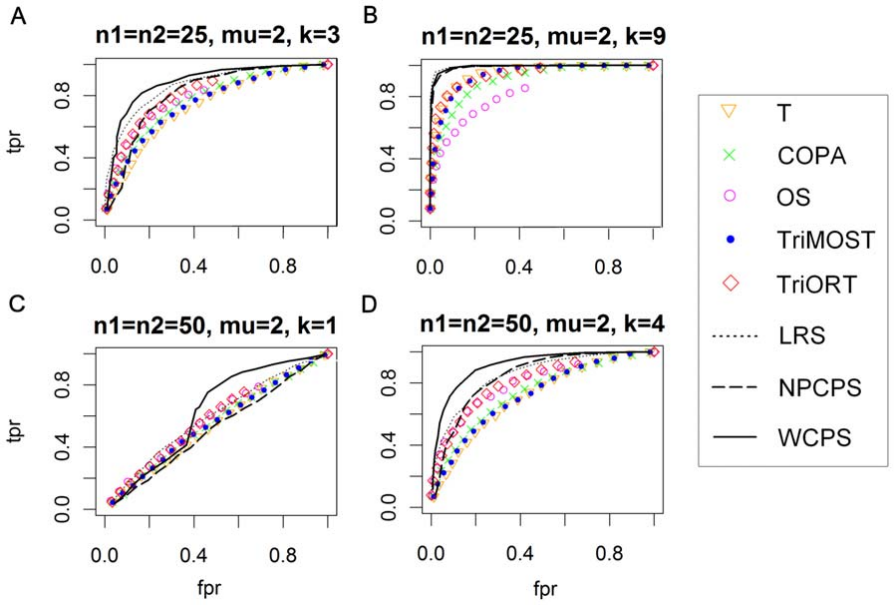
ROC curves of the simulation on data in normal distribution. (A) *n*_1_ = *n*_2_ = 25, μ = 2, *k* = 3. (B) *n*_1_ = *n*_2_ = 25, μ = 2, k = 9. (C) *n*_1_ = *n*_2_ = 50, μ = 2, k = 1. (D) *n*_1_ = *n*_2_ = 50, μ = 2, k = 4. The x-axis is FPR, and the y-axis is TPR. The significance level α = 0.01 for WCPS and NPCPS. Larger area under ROC curves indicates better sensitivity and specificity. An ROC curve along the diagonal line indicates random-guess.

**Figure 2:**
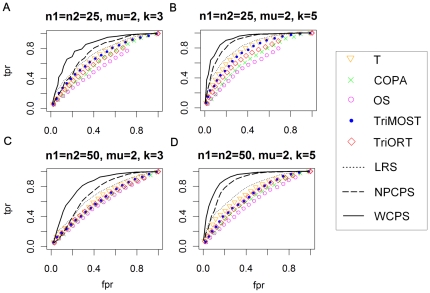
ROC curves of the simulation on data in skew-normal distribution. (A) *n*_1_ = *n*_2_ = 25, μ = 2, *k* = 3. (B) *n*_1_ = *n*_2_ = 25, μ = 2, k = 5. (C) *n*_1_ = *n*_2_ = 50, μ = 2, k = 3. (D) *n*_1_ = *n*_2_ = 50, μ = 2, k = 5. The x-axis is FPR, and the y-axis is TPR. The significance level α = 0.01 for WCPS and NPCPS. Larger area under ROC curves indicates better sensitivity and specificity. An ROC curve along the diagonal line indicates random-guess.

The proposed method and other seven methods as comparison were then applied to two types of dataset, one in normal distribution and the other in skew-normal distribution, and each type contained several datasets with different μ, *n* and *k*. The other seven methods are NPCPS, LRS, TriMOST, TriORT, COPA, OS and T-statistics. The AUC of ROC analysis on both types of dataset is summarized in Table 4 and Table 5, and the ROC in Fig. 1 and Fig. 2, respectively.

**Table 4:** AUC of ROC curves of the simulation on data in normal distribution.

Data Parameter			AUC							
n	μ	k	WCPS	NPCPS	LRS	TriMOST	TriORT	COPA	OS	T
50	2	3	0.87	0.79	0.85	0.73	0.81	0.75	0.78	0.72
50	2	5	0.92	0.88	0.92	0.81	0.86	0.86	0.81	0.81
50	2	9	0.98	0.97	0.97	0.92	0.94	0.88	0.81	0.93
100	2	1	0.61	0.50	0.58	0.54	0.58	0.54	0.58	0.53
100	2	4	0.89	0.82	0.83	0.70	0.80	0.72	0.80	0.70
100	2	9	0.97	0.96	0.96	0.84	0.94	0.89	0.90	0.85
50	1	6	0.75	0.72	0.74	0.70	0.64	0.63	0.59	0.70
50	1	9	0.81	0.79	0.80	0.76	0.71	0.67	0.61	0.78
50	1	14	0.89	0.89	0.89	0.88	0.80	0.71	0.61	0.89
100	1	6	0.74	0.70	0.69	0.62	0.62	0.59	0.59	0.64
100	1	9	0.80	0.78	0.75	0.68	0.67	0.63	0.63	0.70
100	1	15	0.89	0.88	0.86	0.81	0.78	0.72	0.67	0.83
**Mean AUC**			0.84	0.81	0.82	0.75	0.76	0.72	0.70	0.76

**Table 5:** AUC of ROC curves of the simulation on data in skew-normal distribution.

Data Parameter			AUC							
n	mu	k	WCPS	NPCPS	LRS	TriMOST	TriORT	COPA	OS	T
50	2	3	0.81	0.72	0.70	0.66	0.62	0.55	0.56	0.66
50	2	5	0.90	0.86	0.80	0.74	0.70	0.64	0.60	0.76
50	2	9	0.95	0.95	0.90	0.85	0.78	0.64	0.57	0.88
100	2	3	0.80	0.69	0.66	0.59	0.60	0.57	0.57	0.62
100	2	5	0.89	0.84	0.74	0.66	0.66	0.61	0.59	0.69
100	2	9	0.96	0.95	0.86	0.77	0.75	0.67	0.63	0.82
50	1	6	0.68	0.67	0.65	0.65	0.61	0.56	0.55	0.69
50	1	9	0.74	0.75	0.68	0.72	0.63	0.57	0.55	0.75
50	1	14	0.82	0.83	0.71	0.80	0.67	0.59	0.55	0.83
100	1	3	0.61	0.59	0.57	0.55	0.55	0.53	0.54	0.58
100	1	6	0.67	0.65	0.61	0.63	0.59	0.55	0.55	0.64
100	1	20	0.88	0.89	0.73	0.82	0.68	0.61	0.58	0.84
**Mean AUC**			0.81	0.78	0.72	0.70	0.65	0.59	0.57	0.73

Results show that the proposed method had larger AUC than the other methods, more significantly when *k* was smaller. Generally, change-point based methods, namely WCPS, NPCPS and LRS were better than the percentile-based methods in terms of ROC in the simulation study, while WCPS had the best performance; among the percentile based methods, T-statistic was very effective, while TriORT and TriMOST were better than the other two methods in terms of ROC.

The simulation result proved that by adding a weight to the original function, the proposed method becomes more sensitive to smaller *k*.

### DGE detection in microarray data of breast-cancer

#### Result on Dataset I

Dataset I contains microarray data of 49 samples from breast cancer tissues as described in the Material and Methods section. Based on the previous experiment result, among the 5293 valid and unique genes of the dataset, NPCPS (*C*(0.05) = 1.628) yielded a detecting result of 1598 DGE genes and 17 out of 36 top ranked genes were reported as relevant to breast cancer or other known cancers. By applying the proposed method to the same dataset, for *C*(0.05) = 1.628, there were 2279 DGE genes being detected (1258 over expressed genes and 1021 under expressed genes, respectively); for *C*(0.05) = 1.358, there were 3974 DGE genes being detected (2230 over expressed genes and 1744 under expressed genes, respectively). All the top 50 ranked genes were reported as cancer-relevant.

Among the recognized differentially expressed genes, most of them have been reported as involved directly with cancer in published papers, such as AGER, MAPK14, etc. Some genes themselves have not yet been reported, but their related genes, proteins, or behaviors have been reported as cancer-relevant, such as DGKD (EGFR and DAG related, ranked 481) [20]. Some of the genes with higher *D_n_* statistic are suspected as participants of cancer cell lines. For example, gene CCDC130 (ranked 384) is potentially cancer relevant and currently under research in order to reveal the characterization of CCDC130 in cancer cell signaling [21]. Gene ranked in the first 500, such as AHDC1 (ranked 159), LIG3 (ranked 409), DMD (ranked 75), have not yet been reported formally as cancer-relevant. However, given the significant difference between cancer and normal group, it is reasonable to assume there is high possibility that these genes might participate in cancer development.

**Figure 3:**
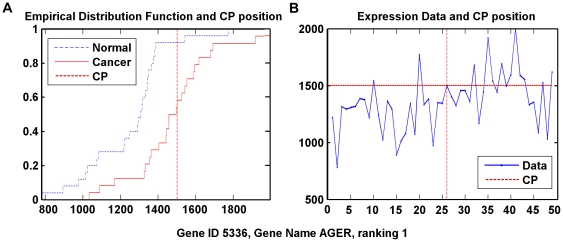
Data distribution of Gene AGER, ranked 1st by WCPS. (A) Empirical distribution functions of cancer and normal group, respectively, with the expression value at the change-point. (B) Expression data by samples, as well as expression value at the change-point.

**Figure 4:**
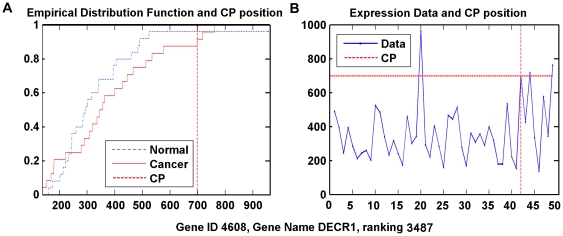
Data distribution of Gene DECR1, ranked 3487th by WCPS. (A) Empirical distribution functions of cancer and normal group, respectively, with the expression value at the change-point. (B) Expression data by samples, as well as expression value at the change-point.

Some of the top 50 genes are listed in Table 6 with the cancer-relevant description [22]–[53]. The data distributions of two typically ranked genes are in Fig. 3 and 4. It is clear that the estimated change point could locate the actual changing point in the gene expression data. Particularly, the cancer samples that are ‘more overly expressed’ than the sample on the change point could be recognized as located in the area specified by the red dashed lines of CP.

**Table 6:** Cancer-related description of top-ranked genes.

Rank	Gene	Description
1	AGER	Strong expression is seen in cells at the invasive edge of tumors and correlates with invasion and lymph node metastasis [22]
2	GP1BB	Different histological types of lung cancer may be distinguished from normal tissue based on differential DNA methylation of GP1bbeta [23]
3	PDE4B	The phosphodiesterase PDE4B limits cAMP-associated PI3K/AKT-dependent apoptosis in diffuse large B-cell lymphoma [24]
4	MAPK14	The expression of p-p38 and uPA was negatively correlated to prognosis of breast cancer [25]
5	SMARCA2	Encodes BRM in the SWI/SNF chromatin-remodeling complex. SWI/SNF related and loss of SWI/SNF-mediated transcriptional activation increases DNA methylation in cancer cells [26]
6	TCF3	Protein TCF3 no longer binds DNA when modified by a phosphate, making Phosphorylated TCF3 a new diagnostic marker for cancer [27]
7	NCSTN	NCSTN coded protein is a subunit of γ-Secretase compound, which is related to Notch signaling, a pathway found dysregulated in many cancers [28]
8	C9	Upregulation of plasma C9 protein in gastric cancer patients [29]
9	SCARB2	SCARB2 and CSNK1 double negative mRNA expression seems to be predictive of the presence of non-compromised lymph nodes in oral squamous cell carcinoma [30]
10	BMP1	BMP molecules have further been shown to have an impact on the biological behaviour of breast cancer cells [31]
11	MEF2A	Mediates synergistic transcriptional responses to the CaMK and MAPK signaling pathways by signal-dependent dissociation from histone deacetylases [32], which regulate the expression and activity of numerous proteins involved in both cancer initiation and cancer progression [33]
12	MYOG	Terminal myogenesis switches off cell proliferation and migration, hence, the promotion of rhabdomyosarcoma differentiation should antagonize tumor growth and metastasis [34]
13	RPL36A	Over-expression of RPL36A is associated with cellular proliferation in hepatocellular carcinoma [35]
14	SLC5A5	NIS expression is prevalent in breast cancer brain metastases and could have a therapeutic role via the delivery of radioactive iodide and selective ablation of tumor cells [36]
15	JAG1	Associated with a basal phenotype and recurrence in lymph node-negative breast cancer [37]
16	MMP11	Expression reflects the stages of tumor differentiation and LNM of breast cancer [38]
17	NEFL	Neurofilament proteins are markers for neuroendocrine tumors [38]
18	SLC4A2 (AE2)	AE2 might be associated with gastric carcinogenesis and the achlorhydria experienced by gastric cancer patients [40]
27	MYL1	Myosin VI is critical in maintaining the malignant properties of the majority of human prostate cancers diagnosed today [41]
28	IGHD	Immunoglobulin D enhances the release of tumor necrosis factor-alpha [42]
29	ZNF131	Repressor of ERalpha signaling [43]
30	RBBP6	Involvement of RbBP6 gene and apoptosis in the pathogenesis of lung cancer [44]
31	IQGAP1	IQGAP1 plays a critical role in colon cancer cell invasion, and therefore diffuse and high expression of IQGAP1 predicts poor prognosis in patients with colorectal carcinoma [45]
35	UNC119	UNC119 is required for G protein trafficking in sensory neurons [46], while G protein signaling is involved in tumor growth and angiogenesis [47]
38	PTPRR	The protein tyrosine phosphatase receptor type R gene is an early and frequent target of silencing in human colorectal tumorigenesis [48]
39	UBB	Essential mediator of trichostatin A-induced tumor-selective killing in human cancer cells [49]
40	MGST2	Microsomal glutathione Stransferase II. Glutathione plays a critical role in cellular mechanisms that result in cell death [50]
44	ACAP1	ACAP1 is a GTPase activating protein specific for Arf6 [51], which is required in breast cancer invasive activities [52]
47	NAT6 (FUS2)	Function of NAT6 plays an important role in cancer as the gene maps to the chromosomal region 3p21.3, which includes at least one tumor suppressor gene [53]

**Figure 5:**
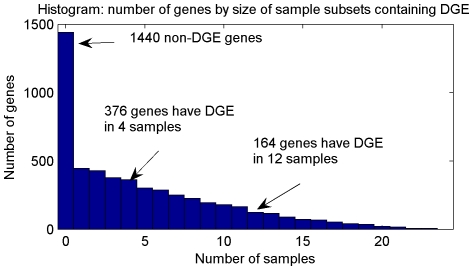
Histogram: number of DGE genes by size of sample subsets containing DGE. There are 1440 non-DGE genes; 376 genes have DGE in 4 cancer samples; 164 genes have DGE in 12 cancer samples.

The number of DGE samples of each gene is calculated and the corresponding histogram of detected DGE genes is displayed in Fig. 5. For example, there are 1440 non-DGE genes; 376 genes have DGE in 4 cancer samples; 164 genes have DGE in 12 cancer samples. Given the cancer group size 24, this histogram demonstrates that DGE may only exist in cancer subgroup.

**Figure 6:**
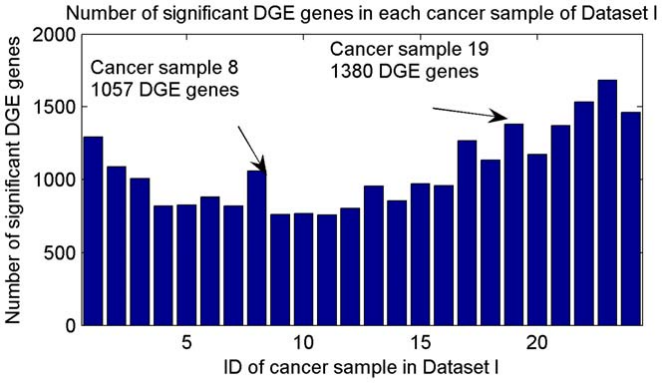
Number of significant DGE genes in each cancer sample of Dataset I. There are 1057 DGE genes in cancer sample 8; 1380 DGE genes in cancer sample 19; 1682 DGE genes in cancer sample 23.

Accordingly, the number of differentially expressed genes in each cancer sample is calculated as shown in Fig. 6. For example, there are 1057 DGE genes in cancer sample 8, 1380 DGE genes in cancer sample 19, and 1682 DGE genes in cancer sample 23.

#### Result on Dataset II

As described in the Section of Material and Methods, Dataset II contains microarray data of 42 samples of 12576 genes, 18 samples of histologically normal (HN) epithelium from breast cancer patients, 6 samples of high-risk prophylactic mastectomy (PM) patients, and 18 samples of reduction mammoplasty patients. After applying WCPS to the dataset, when threshold is 1.358, there are 9793 over-high expressed gene and 190 over-low expressed genes, respectively; when the threshold is 1.628, the over expressed genes reduced to 867 over-high and 10 over-low, respectively. Apparently, this dataset contains majorly over-high expressed genes. Among the 50 top-ranked genes, 43 genes have been clearly reported as relevant to human cancer. Among the rest 6 genes, third-ranked gene AP000944.1 is a lincRNA and long non-coding RNA has drawn the research attention of its functional role in human cancer [54]; CENPM gene itself are not yet reported as cancer-relevant, but inappropriate expression of the centromere proteins CENP-A and CENP-H could be a major cause of chromosomal instability that has been recognized as a hallmark of human cancer [55]; 50-ranked gene HPN cooperates with MYC in the progression of adenocarcinoma in a prostate cancer mouse model [56].

NPCPS was also applied to this dataset and yielded 2564 and 337 differentially expressed genes with threshold 1.358 and 1.628, respectively.

**Figure 7:**
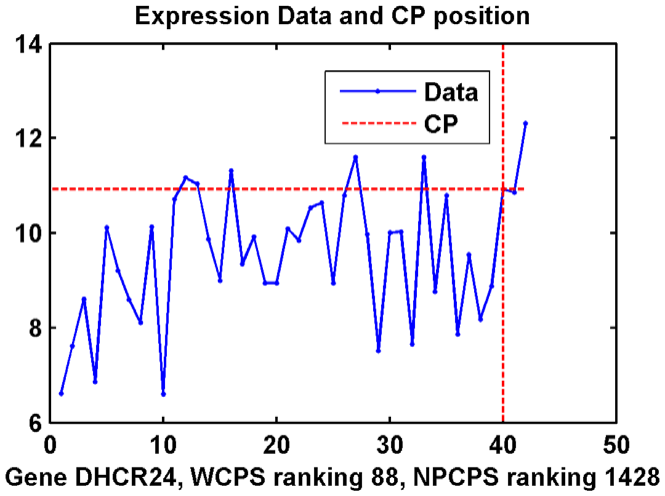
Data distribution and CP of Gene DHCR24 in Dataset II. (A) Empirical distribution functions of cancer and normal group, respectively, with the expression value at the change-point. (B) Expression data by samples, as well as expression value at the change-point.

**Figure 8:**
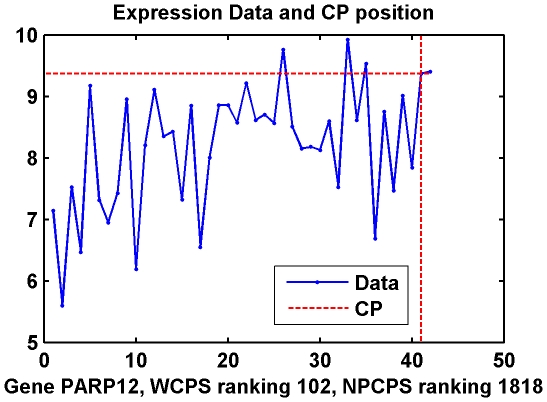
Data distribution and CP of Gene PARP12 in Dataset II. (A) Empirical distribution functions of cancer and normal group, respectively, with the expression value at the change-point. (B) Expression data by samples, as well as expression value at the change-point.

WCPS detected much more differentially expressed genes compared with NPCPS. Moreover, the rankings between these two methods are only about 50 percent relevant. WCPS successfully recognized genes that are lower ranked or ignored by NPCPS. Fig. 7 and Fig. 8 show expression data of several such genes.

**Figure 9:**
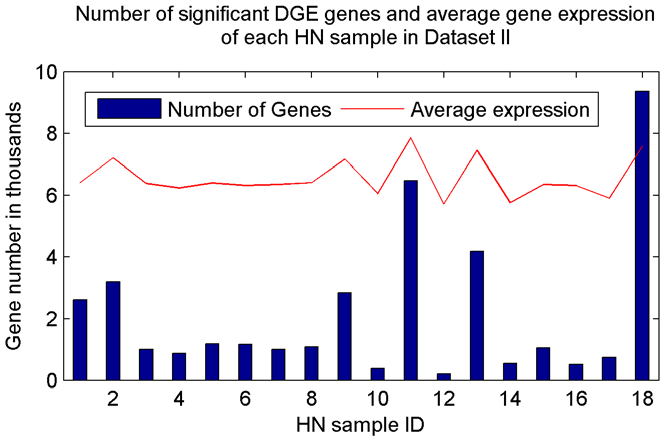
Number of significant DGE genes in each HN sample of Dataset II. HN sample 11 and 18, two ER+ breast cancer patient samples, have more than 6000 differentially expressed genes. HN sample 1, 2, 9, three ER− breast cancer patient sample and 13, an ER+ patient sample have more than 2000 differentially expressed genes.

**Figure 10:**
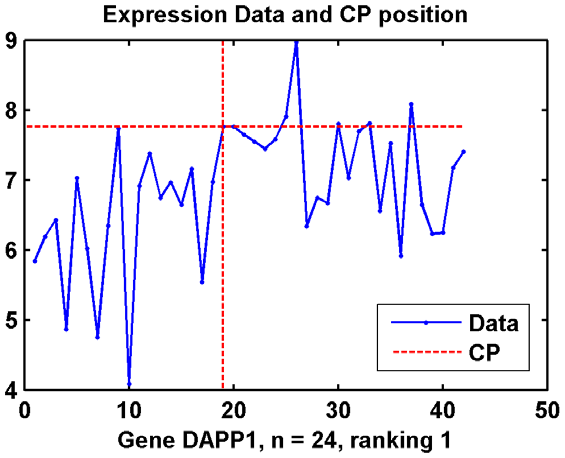
Data distribution and CP of Gene DAPP1 in Dataset II. Gene expression of PM samples are not only over-expressed compared with the RM samples in case group, but also generally higher than the 18 HN samples from breast cancer patients.

Fig. 9 illustrates the total number of DGE genes in each HN sample. HN sample 11 and 18, two ER+ breast cancer patient samples, have more than 6000 differentially expressed genes. HN sample 1, 2, 9, three ER− breast cancer patient sample and 13, an ER+ patient sample have more than 2000 differentially expressed genes. Fig. 10 is the top ranked gene by WCPS.

The 6 PM samples are from high-risk women and, as in the work by Graham et al., gene expression in histologically normal epithelium from breast cancer patients and from cancer-free PM patients shares a similar profile [57]. Therefore, we also tested the dataset consisted of 6 PM samples as the case group and 18 RM samples as the control group. As a result, when threshold *C*(0.05) = 1.358, there are 7344 over-expressed genes and 79 under-expressed genes, respectively. Fig. 10 shows one of the top-ranked genes, in which the gene expression of PM samples are not only over-expressed compared with the RM samples in case group, but also generally higher than the 18 HN samples from breast cancer patients.

**Figure 11:**
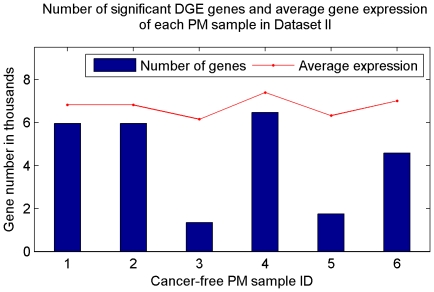
Number of significant DGE genes in each PM sample of Dataset II. PM sample 1, 2, 4, and 6 have significantly more DGE genes compared with PM sample 3 and 5. This result corresponds to the average expression of the 12576 genes from the 6 samples.

Fig. 11 summarizes number of DGE genes in each PM samples. PM sample 1, 2, 4, and 6 have significantly more DGE genes compared with PM sample 3 and 5. This result corresponds to the average expression of the total 12576 genes from the 6 samples.

## Materials and Methods

### Change-point in gene expression

The method we proposed here inherited the definition of change-point as described in NPCPS [18]. Consider gene expression value as a sequence of independent variables as below:
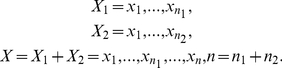
(1)


Here, *X*_1_ contains expression values of normal samples in known distribution function *F*_1_ (*x*), and *X*_2_ contains expression values of cancer samples. Over or under expression values in *X*_2_ would result in a change point in *X*. The existence of change point is evaluated by a modified Kolmogorov statistic (K-statistic), which indicates the distance between two distribution functions. Suppose *F*_1_^−1^ (*y*) is the inverse function of *F*_1_(x), which is defined as
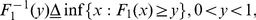
(2)where y is a variable increasing with a fixed step that is subject to user's selection. Then, the testing procedure is defined as
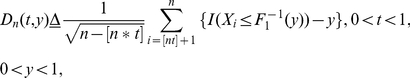
(3)where [n_*_ *t*] means round toward negative infinity. *X* has a change point when 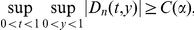 where 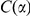 is the critical value and α is the significance level, while typical values include *C*(0.05) = 1.358 and *C*(0.05) = 1.628.

### Weighted Change-point Statistic

**Figure 12:**
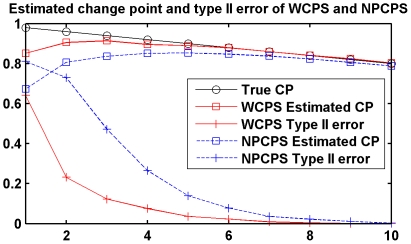
Estimated change point and type II error of NPCPS. NPCPS is not very sensitive to the right bound in terms of type II error and estimated CP position. Both estimated change point and type II error of WCPS show better results compared with NPCPS.

**Figure 13:**
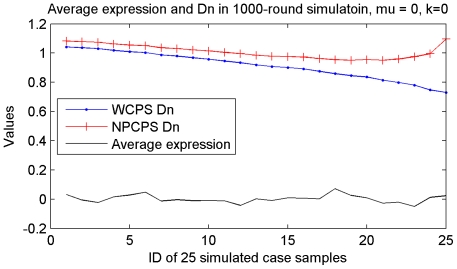
*D_n_* values of each sample in a gene expression profile without DGE. In simulated data without any DGE, *D_n_* value has a descending trend when approaching the right bound. Weighted *D_n_* moderately compensated the descending trend of *D_n_* statistic.

The aim of NPCPS is to find the largest *D_n_* and check if the value exceeds the threshold, while the position of the largest *D_n_* value indicates the most significant changes in the expression profile of a single gene. According to the ROC curves obtained from simulation study [18], NPCPS was more than 99% correct when for a single gene there are more than 9 samples that contain DGE. However, NPCPS is not very sensitive to the right bound as shown in Fig. 12. When there is only a small subset of cancer group, especially when k<5, NPCPS would have inadequate *D_n_* values and consequently would not always report the existence of change point. Fig. 13 illustrates the descending trend of *D_n_* value. When there is no simulated DGE added to the normally distributed data, *D_n_* function shows a descending curve.

Therefore, in order to enhance the right-bound sensitiveness, it is reasonable to assume that by adding a proper weight function to the original function, the *D_n_* statistic could be adequately compensated even if the change occurs in the last few data points. Apparently, the goal of the weight function is to moderately compensate the right end of the *D_n_* statistic to avoid a rigid positive result, while keeps the *D_n_* value on the left end as well as in the middle as much as possible, which would resemble a function similar to 1/x. Besides, as *D_n_* is a step function, the weight function should also have the same step as *D_n_* statistic.

**Figure 14:**
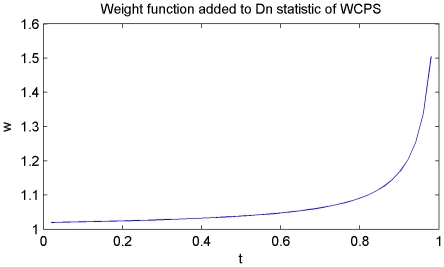
Weight function in WCPS. The ascending curve would compensate the descending trend of original *D_n_* statistic.

The weight function as in Fig. 14 is as follows:
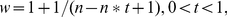
(4)and the weighted *D_n_* is defined as
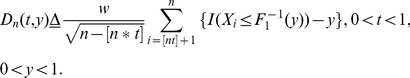
(5)


The weighted *D_n_* function demonstrated better response to small subset that has DGE as shown in Fig. 12. Both estimated change point and type II error of WCPS show better results compared with NPCPS. Besides, from Fig. 13 we can see that adding a weight function does not give an unreasonable rise to the right bound when there is no DGE in any samples of the simulated data.

### Experiment on Breast cancer microarray dataset

Two datasets were tested in the experiment. One microarray dataset (referred to as dataset I) of breast cancer [58], the same dataset used in [18] includes 49 samples all from cancer tissues, with different status of lymph node (LN) and estrogen receptor (ER), i.e. LN+ER−/LN+ER+/LN−ER+/LN−ER−. As the negative-lymph-node breast cancer is categorized as early stage breast cancer, these 49 samples could be categorized into two types: 25 samples with negative lymph node as the normal samples and 24 samples with positive lymph node as the cancer samples, respectively. Besides, gene expression profile of 7129 genes in the samples was obtained through annotation package hu6800 [59]. Probes of genes obsolete in NCBI gene bank were deleted; for multiple probes mapping to the same gene, only the probe that corresponded to the largest *D_n_* was kept. These two steps resulted in a total 5293 genes. This dataset was tested by all methods mentioned in simulation study. Before applied to LRS, COPA, TriMOST, TriORT, OS, and T-statistics, the gene expression values were first normalized. Before applied to WCPS, the expression values in cancer group were sorted in ascending order for each gene.

The other one (referred to as dataset II) is a 42-sample dataset obtained on platform Affymetrix Human Genome U133A Array. The samples contains 3 subsets: 18 samples of normal breast epithelia from reduction mammoplasty patients (RM sample); 18 samples of histological normal breast epithelia from 9 ER+ and 9 ER− breast cancer patients (HN samples); and 6 samples of histologically normal breast epithelium from prophylactic mastectomy patients (PM samples) [57]. 18 RM samples and 6 PM samples were considered as the control group, while the 18 HN samples were the case group in the original article. This dataset was tested by WCPS.

For method NPCPS, LRS, TriMOST, TriORT, COPA, OS and T-statistic, the genes were ranked according to the different statistic in descending order. Genes ranked in the top indicated higher degree of DGE.

For WCPS, change-point was determined by weighted *D_n_* statistic. Genes with weighted *D_n_* larger than 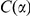 were recognized as having DGE. Specially, for detecting result under 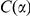 = 1.358 and based on the type of DGE (over high or over low), sample values that exceed the expression value at the change-point could be identified on single gene level. This would result in an array containing binary values of 0 or 1, where 0 indicates non-DGE sample and 1 indicates significant DGE sample. Therefore, for all genes in a dataset, these arrays could be combined to construct a matrix. Based on the matrix, the DGE genes contained in each cancer sample, or the size of DGE cancer sample subset could be calculated.
